# Implementation and experiments for interactive lyrics transcreation system

**DOI:** 10.1186/s42492-020-00053-x

**Published:** 2020-07-30

**Authors:** Ayano Nishimura, Takayuki Itoh

**Affiliations:** Ochanomizu University, 2-1-1 Otsuka, Bunkyo-ku, Tokyo, 1128610 Japan

**Keywords:** Lyrics transcreation, Score visualization, Interactive system

## Abstract

The evolution of the Internet has enabled us to enjoy the music created in various countries. But still, it is often difficult to understand the lyrics written in foreign languages. Professional translators have published many international songs with lyrics that fit the melody so that the ordinary people can enjoy the lyrics of such international songs. This paper discusses lyrics transcreation into the Japanese language. Also, the paper presents an interactive visual lyrics transcreation system and describes the details of its implementation. This system allows users to select temporary lyrics from a set of tentative translations and then freely modify the lyrics with a real-time visualization mechanism. We also propose a lyrics translation algorithm that solves an essential problem of lyrics translation into Japanese. In this study, we interviewed two experts regarding problems with lyrics translation and received reviews of our presented system. We also conducted preliminary experiments with 19 participants to determine the best combination of user interface components for our system. We performed additional user experiments inviting 12 participants to compare lyrics transcreation using the presented system to manual lyrics transcreation. Lyrics transcreation by the presented system brought better results against those of manual transcreation.

## Introduction

The Internet has made us easier to access international songs and read lyrics written in foreign languages. But still, it is often difficult to interpret foreign lyrics. Literal or free translations are often accompanied by lyrics in the original languages; however, the translated sentences may not fit the melody and may therefore be difficult to sing.

However, there are many translated international songs whose translated lyrics fit the original melodies. Not only popular songs, such as children’s songs, but also songs from musicals and operas have been translated. The task of lyrics translation requires an understanding of the original language, musical phrases, rhythm, and pronunciation. This task is thus left to professionals. In particular, translating songs from a musical or opera is often difficult, as it involves multiple songs and requires an understanding of the story’s flow through all acts. Furthermore, according to some studies [1, 2] the language of the lyrics affects the song rhythm.

We have developed an interactive visual lyric transcreation system for translating lyrics produced in foreign languages into the languages of users (English to Japanese in our implementation) [3] to deal with the above challenges. Transcreation refers to the process of adapting a text from an original language to a target language while maintaining the intent, style, tone, and cultural context.

Our system visualizes a user’s edits in real-time on a score. The real-time visual reflection of the user’s edits on the musical score makes it easier to read the transcreated lyrics and melodies than using conventional handwritten transcreation. This mechanism also improves the efficiency of transcreation. The presented system helps users who enjoy singing songs but are not familiar with foreign languages. The system can also help organizations that provide songs to singers, such as amateur theater companies that play operas in their native language. In addition, we expect the transcreation of foreign songs using the presented system can help develop a diverse musical culture.

We interviewed two lyrics translation experts regarding the problems of lyrics translation as well as their impressions of our presented system. We also recruited 19 participants for a preliminary experiment to select the best components of the visual user interface. We then comparatively evaluated the presented system through an experiment with 12 participants. In this paper, we describe the presented system and its implementation as a web application, and discuss the experimental results.

### Problems with translation of lyrics into Japanese

Sakufu Kondo was one of the pioneer of lyrics translation into Japanese [4]. Kondo emphasized the original meaning and translation of prosody as much as possible. Similarly, our presented system attempts to automatically reproduce the meaning and prosody of the original language to the largest possible extent.

In this section, we discuss the problems associated with translating lyrics into Japanese. Languages are typically categorized into three classes: stress-timed, syllable-timed, and mora-timed languages. Japanese belongs to mora-time languages [5, 6]. In this study, we define one mora as a vowel, a consonant followed by a vowel or contracted sound. Contracted sounds are indicated when certain ‘I’ vowel characters are followed by smaller sized “ya(ゃ)”, “yu(ゅ)”, and “yo(ょ)”. The number of morae usually increases when translating from non-mora-timed languages into Japanese. We need to prevent an increase in the number of morae, and therefore we need to abbreviate the meaning of the original lyrics [7].

We interviewed two experts with regard to the problems of lyrics translation. According to one of the experts, matching lyrics translated into Japanese with the melody is difficult because the number of sounds per word increases after translating the lyrics into Japanese, assuming that the original language is English and there is one word per sound. In particular, it is difficult to select appropriate Japanese words for a melody when the last important word of a phrase has a high note or long tone. In addition, the article at the beginning of a phrase is often an anacrusis. The lyrics appear poorly-transcreated if an anacrusis is substituted by an exclamation word when important words follow the anacrusis. However, it is often possible to shorten a sentence because sentences in Japanese can be completed without subject words, such as ‘I’. In addition, Japanese people are used to understanding ambiguous sentences. The second expert mentioned that only one mora is usually assigned to one note when translating lyrics into Japanese. This expert was thus concerned whether a word could be properly recognized when sung as a sound. This problem is largely influenced by Japanese intonation, the highs and lows of the melody, and the position of the rests.

It is also necessary to consider suitable pronunciation for singing [8] in Japanese. From the viewpoint of a syllable, the Japanese syllabic nasal, double consonants, a long vowel, and double vowels may not be included in the number of morae by being sung together with the previous pronunciation. This affects the sense of rhythm while singing.

In addition, ‘a’ and ‘o’ are resounding vowels, whereas ‘e’, ‘I’ and ‘u’ are not when singing in Japanese. We may therefore need to consider the trade-off between the importance of the meaning of words and the effect of given vowels on singing during translation tasks.

The problems associated with English to Japanese lyrics translation techniques are summarized as follows:

(1) The number of morae typically increases when translating into Japanese;

(2) The meaning of the original language must be selected;

(3) When considering rhythm and syllables, it is necessary to determine whether the pronunciation is a single mora;

(4) Vowels for certain notes must be limited for musical reasons.

Naturally, a variety of translations can be created even by manual translation. In addition, it may be difficult to select the best translation from a set of candidate translations automatically generated by a system. Therefore, users should be able to adjust parameters based on their preferences and edit the scores generated by the system. In this study, we focused on Problem 1 and sought a solution.

### Related work

The translation of foreign songs into Japanese has been discussed in a number of studies. Matsuda focused on lyrics translator Sakufu Kondo and discussed how to translate foreign songs, such as operas and musicals, into Japanese [4, 7]. Miyamoto addressed the problems of lyrics translation from the perspective of Japanese pronunciation, accent, and rhythm [9]. Although these studies address lyrics translation problems, to the best of our knowledge, there have been no studies on the development of lyrics translation systems that solve these problems.

There have been automatic and interactive techniques on lyric and melody generation. For example, “Tra-la-lyrics 2.0” [10] automatically generates song lyrics in Portuguese based on a melody. ‘Orpheus’ is an automatic music composition system that generates melodies from lyrics provided by users and considers Japanese prosody [11]. The target of our study is to develop an interactive lyrics transcreation system. A component of the system features an algorithm for modifying the melody according to the lyrics and generating tentative translations.

‘Hafez’ provides an interactive environment for poetry generation without melodies while applying a recurrent neural network featuring a finite-state acceptor [12]. Another lyrics creation system ‘pâtisier’ suggests lyrics considering the numbers of morae and vowels [13]. ‘LyriSys’ supports the creation of the lyrics while considering the comprehensive structure of lyrics including verses and choruses [14].

A number of studies, including those mentioned above, have aimed to automatically generate lyrics or melodies. However, our goal is to transcreate lyrics while maintaining form and content under the assumption that both the lyrics and melody are provided in advance.

The translation of film subtitles has similar problems to lyrics translation. Film subtitles are displayed for a short time; therefore, audiences only have a limited time to perceive and understand them. We believe that this problem is similar to the limitation of the number of morae in lyrics translation. Various machine translation systems have been designed for document translation, and a system for translating closed captions has also been developed [15]. In addition, the characteristics of machine translation for film subtitles have been described [16].

Sentence compression techniques discussed in natural language processing communities relate to the reduction of the number of morae in translated lyrics. Yamamoto [17] proposed a partial machine translation method that translates partial expressions. This method inputs a partial expression and a source language sentence and outputs a concise translation centered on the partial expression. There have also been several commercial systems on short and simple sentence generation such as the electrical bulletin board of Shinkansen trains [18].

## Methods

### System architecture

**Fig. 1:**
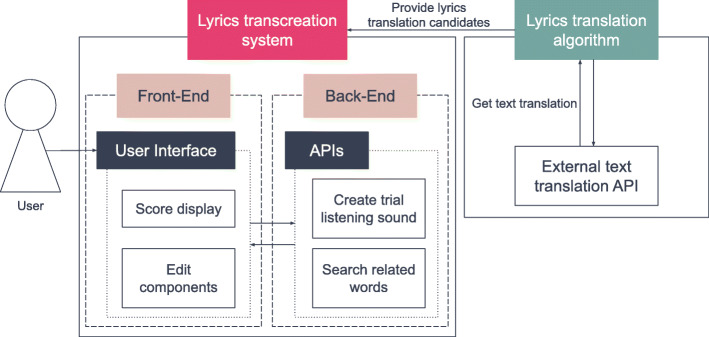
System architecture

Figure 1 illustrates the system architecture implemented in this study. Our implementation can be divided into two components: a lyric translation algorithm and a lyric transcreation system.

The presented system translates the original lyrics using an external application programming interface (API), and then processes the translated text into the lyrics format using the lyrics translation algorithm.

The lyrics transcreation system is deployed with back-end and front-end servers. Singing voice synthesis software and a related words model are installed on a backend server separately from the front-end server. The front-end server provides a user interface for partial modification of phrases. Users can freely edit the lyrics in the temporary translation on the web interface. Figure 1 illustrates the functions that were adopted through a preliminary experiment for selecting the user interfaces.

### Lyric translation algorithm

This section describes the algorithm of lyrics translation and score generation. We adopted a rule-based algorithm, because it is difficult to obtain a large parallel corpus of melodies for a neural network.

**Fig. 2:**
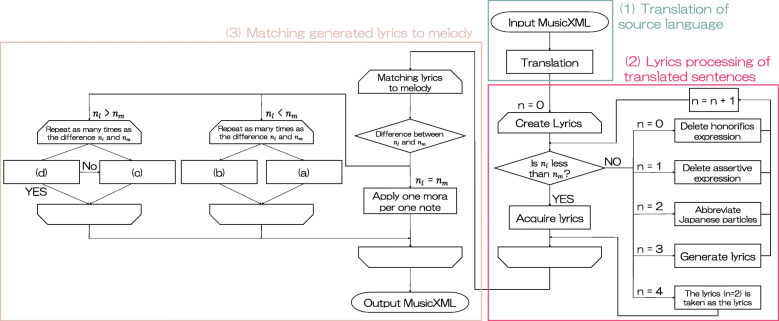
Processing procedure of lyrics transcreation (arranged from ref. [3])

Figure 2 shows the processing flow. The processing flow consists of the following three components: (1) translation from the original language, (2) modification of translated sentences, and (3) matching between generated lyrics and the melody.

#### Translation of the original language

This component acquires machine-translated sentences and the number of musical notes of each phrase.

This system uses the Microsoft Translator Text API [19] to obtain sentences of lyrics translated from English to Japanese. The sentences are described in MusicXML while regarding periods, commas, and semicolon delimiters as one phrase. The system treats the number of musical notes in the original score as the number of morae in the original lyrics.

One problem with our implementation is that the machine translation API sometimes fails to translate English sentences; therefore, alphabet words remain in the translated lyrics. In this case, alphabets must be deleted because our current implementation does not support syllable analysis of English sentences.

#### Modification of translated sentences

The presented system modifies the machine-translated lyrics and counts the number of musical notes in this component. The following four operations are applied sequentially if the number of morae of one phrase in a machine-translated sentence is larger than the number of musical notes. Step 1: remove honorific expressions. Step 2: remove assertive expressions. Step 3: abbreviate Japanese particles. Step 4: rearrange the lyrics. This process is applied to all phrases. Here, Japanese syllabic nasal is counted as zero mora. This implementation uses MeCab for morphological analysis [20].

The system executes Step 4 after Steps 1 to 3 if the number of morae is larger than the number of musical notes. We apply the two-gram to rearrange the lyrics to make the number of morae and the number of musical notes equal. We also apply the term frequency-inverse document frequency (tf-idf), a statistical value for measuring the importance of words in a document. This algorithm assigns the word that has the highest tf-idf value as the first word of the phrase. Our implementation applies the corpus [21] created by Project Gutenberg and Aozora Bunko as the reference document while computing the tf-idf value. We also apply the Japanese Wikipedia corpus [22] while rearranging the lyrics.

Step 4 in the lyrics generation algorithm may fail if the number of morae or the first word has troubles. The system applies the sentence modified in Steps 1–3 as the final lyrics in this case and allows it to exceed the number of musical notes.

#### Matching between generated lyrics and the melody

This paper defines three cases between the number of lyric morae *n*_*l*_ and the number of musical notes *n*_*m*_ as follows. Case 1: *n*_*l*_ = *n*_*m*_, Case 2: *n*_*l*_ < *n*_*m*_, and Case 3: *n*_*l*_ > *n*_*m*_. It may occur that the breaks in the melody and the lyrics do not match even if *n*_*l*_ and *n*_*m*_ are equal. For simplicity, we do not address this problem in this study. Instead, we allow it to remain and expect users to edit the machine-translated lyrics and improve the mismatch between breaks. This system does not suggest to modify the melody if *n*_*l*_ and *n*_*m*_ satisfy Case 1. Otherwise, for Cases 2 and 3, one of the following algorithms is applied to match the melody and lyrics.

Here, songs in Japanese often allow the stretching of a vowel of one character and singing multiple notes. Here, we define *dur*() as a function of sound duration. In Case 2, when the duration of the *ith* musical note satisfies *dur*(*i*) < *dur*(*i* + 1), we insert a stretch bar ‘ー’ in the *ith* musical note as illustrated in Fig. 2a., the number of morae increases by this step. The first algorithm inserts a stretch bar at the end of the lyrics when all durations of the musical notes are equivalent. The second algorithm combines the note that is the shortest in the phrase with its previous note as illustrated in Fig. 2b. Or, the algorithm may combine the shortest note with its next note if it is impossible to combine with its previous note.

In Case 3, the algorithm divides the longest musical note into two notes that have half the duration. If there are multiple longest nodes, the algorithm selects the one that is closest to the beginning of the phrase. Otherwise, if the note is a dotted note, it is divided into two notes whose lengths are 2:1, as illustrated in Fig. 2c. The second algorithm specifies the duration of the minimum note duration beforehand, and also divides other notes avoiding the notes get shorter than the minimum duration, as shown in Fig. 2d. We define the minimum duration as the eighth note. Even if the algorithm divides the all notes into the minimum duration, the algorithm (c) is applied if *n*_*l*_ becomes larger than *n*_*m*_.

### Preliminary implementation

**Fig. 3:**
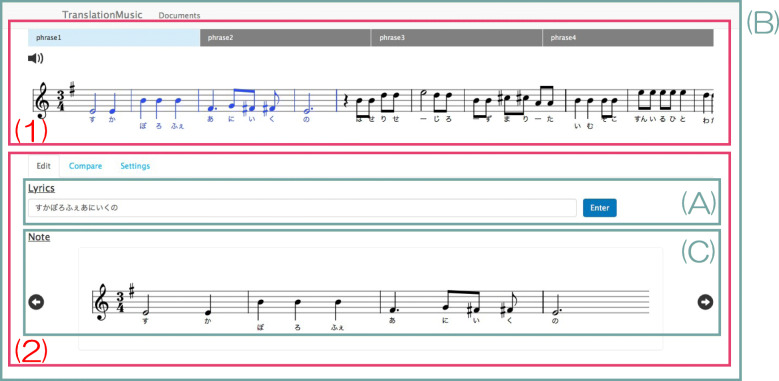
Screenshot of preliminary implementation

Because there have not been sufficient studies on interactive lyrics transcreation, it is necessary to investigate which types of user interfaces are suitable for interactive lyrics transcreation. We implemented a prototype system that can switch functions to create the optimal combination of user interface components, as illustrated in Fig. 3. The user interface is divided into two components: (1) the score and (2) tab functions.

The score component is displayed at the top of the window. A phrase bar indicates the phrase being edited at the top of the score component. Meanwhile, the area for the score marks the target phrase in blue. Users can select phrases while scrolling through the score. In addition, users can listen to their edited songs by pressing the speaker icon in the upper left of the score component. Note that this trial listening function provides the sound source corresponding to the entire song. This function uses the singing voice synthesizer ‘Sinsy’ [23]. Sinsy applies the hidden Markov model and deep neural network to generate voices of singers based on musical scores written in MusicXML.

Three functions are available in the tab component: Edit, Compare, and Settings. The Edit tab features functions that allow users to edit lyrics and notes, while the Compare tab displays the original English lyrics and the edited Japanese lyrics. The Settings tab features buttons to display the application’s help menu and complete the editing of the score.

**Fig. 4:**
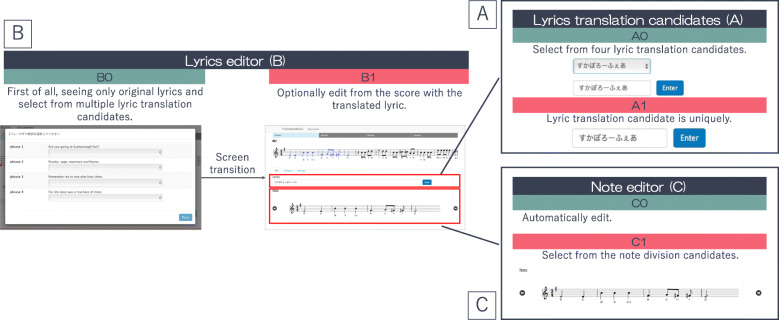
Interface functions. (arranged from ref. [3])

The prototype system has three switchable functions: (A) lyrics translation candidates, (B) lyrics editor, and (C) note editor (Fig. 4). Note that each function has two implementations, denoted ‘0’ or ‘1’. For example, function A’s interfaces are denoted A0 and A1. Among the eight combinations of interfaces, A1 and B0 cannot be combined because the lyrics candidate of A1 is unique, while B0 requires the selection of a single lyrics candidate from multiple candidates. Therefore, excluding these two combinations, the preliminary implementation randomly applies one of six combinations.

#### Lyrics translation candidates

The lyrics translation candidates function has a selection widget and a text field, as illustrated in Fig. 4A. A0 features a selection widget displaying four lyrics as candidates of translations. It displays a set of automatically translated lyrics as the first candidates, while manually translated lyrics are displayed as the second to fourth candidates, in the preliminary implementation. The user interface displays pre-generated lyrics, as the lyrics translation algorithm requires a large computation time to generate lyrics (Step 4) and we do not wish to interactively call the lyrics generation process. The words in the machine-translated sentences that have the largest tf-idf values are presented at the beginning of all lyrics translation candidates. The text field displays the translated lyrics that users selected on the selection widget. It allows the users to freely edit the translated text.

A1 features a text field that initially displays the automatically generated lyrics translation.

#### Lyrics editor

First, B0 displays a modal user interface, as illustrated in Fig. 4B. Here, users can select a translation from the candidates displayed by the selection widget prior to looking at the musical score. A modal close button is enabled when users select lyric translation candidates, and the modal user interface disappears when users press this button. Note that if B0 is combined with A0, the selection widget on the Edit tab displays the lyrics selected in the modal user interface in the initial state.

In contrast, B1 displays the musical score with translated lyrics when users access the system.

#### Note editor

The note editor is used when the number of lyric morae and the number of musical notes are not equal. Here, we use the algorithm presented in matching between generated lyrics and the melodysection.

Note that no user operation is required when applying C0 because musical notes are automatically divided or merged to equalize the number of lyric morae and musical notes. The preliminary implementation matches the lyrics and melody by applying one of the two algorithms randomly.

A user interface for selecting note division is displayed at the bottom of the screen when applying C1, as illustrated in Fig. 4C. This user interface displays the lyrics and melody division candidates in the musical score in the center. Up to two candidates are displayed if *n*_*l*_ > *n*_*m*_ or *n*_*l*_ < *n*_*m*_. Users can switch the display of candidates by pressing the left and right arrow buttons. The selected candidate is reflected in the musical score at the top of the screen when the user clicks the musical score in the center. Note that the phrase color is displayed in red if no melody division is selected.

### Implementation extended after preliminary experiment

**Fig. 5:**
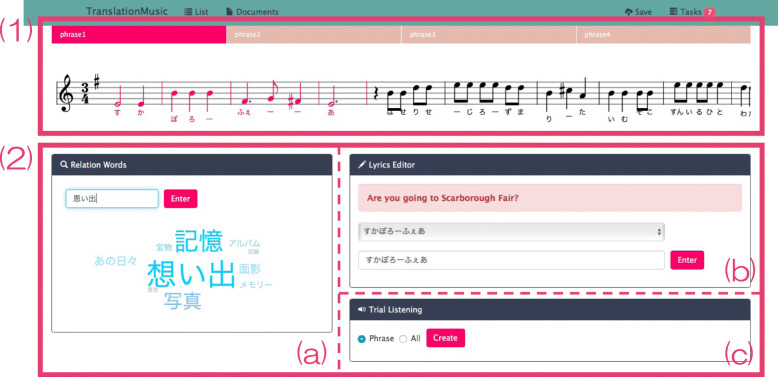
Lyrics transcreation application (arranged from ref. [3])

After the preliminary experiment introduced in preliminary experiment section, we extended our implementation with a musical score editing application for lyrics transcreation that incorporated the results of the preliminary experiments (Fig. 5). As illustrated, the user interface consists of the two components: (1) the score and (2) edit components.

The following functions were updated from the preliminary implementation. Users are now allowed to change phrases, which corresponds to editing the translated lyrics, by scrolling through a score or clicking on the phrase number. The trial listening function is now located in the edit component. Musical notes are typically displayed in black; however, in this system, notes in edited phrases are displayed in pink.

The edit component supports the following tasks: (a) search of related words, (b) edition of translated lyrics, and (c) listening of the edited score. We added a function to search for related words and implemented it by arranging the user interface based on the preliminary implementation.

A task list for lyrics transcreation was also added to the menu bar on the top right. When a user clicks on this menu, the system opens the modal interface of the task. The lists feature gray check icons that turn pink when a user takes a specific action.

#### Search of related words

This component displays related words as a word cloud [24], as displayed in Fig. 5 (2a). When a user writes a word in the text field, the component selects related words based on their cosine similarity displays. It displays the words larger if they have larger cosine similarity values with the input word. Our implementation employs word2vec [25] and a lyrics corpus scraped from piapro [26] to generate a vector space.

#### Edition of translated lyrics

This function comprises a panel, a selection widget, and a text field. The panel displays the original lyrics to compare with the user-edited lyrics.

The function divides melody notes automatically when the number of morae in the input lyrics is greater than the number of musical notes. Two algorithms are applied randomly for note division and visualization of the score.

#### Listening of the edited scores

The extended implementation of the trial listening function allows users to select the listening parts as either a single phrase or the entire song. Here, ‘Sinsy’ is continuously applied to implement this function in the presented system.

## Results and discussions

### Results of lyrics translation algorithm

First, we reviewed the extent to which the number of morae increased or decreased in lyrics translated into Japanese by professionals. We defined *P* as all phrases of the target of the review, *P*_*i*_(*i* = 1, 2, 3…*k*) as one phrase of the target phrases, *P*_*i*_*l* as the number of translated lyric morae, and *P*_*i*_*m* as the number of original musical notes. The difference between the number of original musical notes and the number of translated lyric morae in *P*_*i*_ is called *P*_*i*_*d*. Here, *P*_*i*_*d* can be denoted *P*_*i*_*d* = | *P*_*i*_*m* − *P*_*i*_*l* |. The average increase or decrease of the morae of the translated lyrics in *P* can be defined as follows:

$$ {P}_{avg}=\frac{\sum_{i=1}^k{P}_id}{k} $$

Assuming that x(*n*)(*n* = 1, 2, 3…k) is a permutation of *P*_*i*_*d* in ascending order, the median value can be defined as follows:

$$ {P}_{med}=\left\{\begin{array}{c}\frac{x\left(n+1\right)}{2}\ \left(n= odd\right)\\ {}\frac{x\left(\frac{n}{2}\right)+x\left(\frac{n}{2}+1\right)}{2}\ \left(n= even\right)\end{array}\right. $$

The average and median values were tabulated for 329 professionally translated lyrics phrases, resulting in *P*_*avg*_ = 0.69 and *P*_*med*_ = 0.

We applied the lyrics translation algorithm to four songs: “London Bridge”, “Under the Spreading Chestnut Tree”, “The Itsy Bitsy Spider”, and “Scarborough Fair”. The average and median of all phrases in these songs were *P*_*avg*_ = 1.59 and *P*_*med*_ = 1, respectively. Both the average and median were larger than those of professional translations. The results of each song are presented in Table 1. “Scarborough Fair” resulted in a particularly large difference. This may be due to the complex semantics of the lyrics compared with other songs. “The Itsy Bitsy Spider” exhibited little difference with the professional translation in terms of the numbers: however, the output of the translated lyrics did not make sense for most of the phrases. We obtained positive results for “London Bridge” and “Under the Spreading Chestnut Tree”, whose details are presented below.

**Table 1:** Results of average and median in each song

Song name	*P*_*avg*_	*P*_*med*_
London Bridge	0.4	0.0
Under the Spreading Chestnut Tree	0.75	1.0
The Itsy Bitsy Spider	0.75	0.0
Scarborough Fair	4.75	5.0

**Fig. 6:**
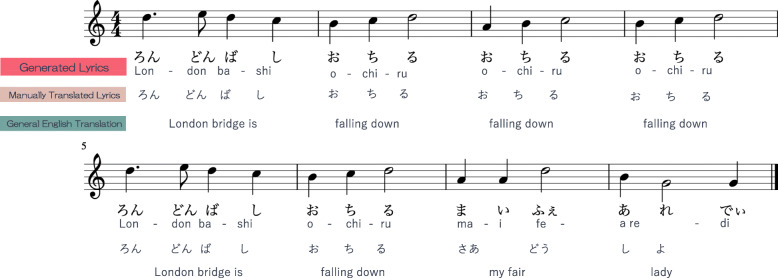
“London Bridge”.

**Fig. 7:**
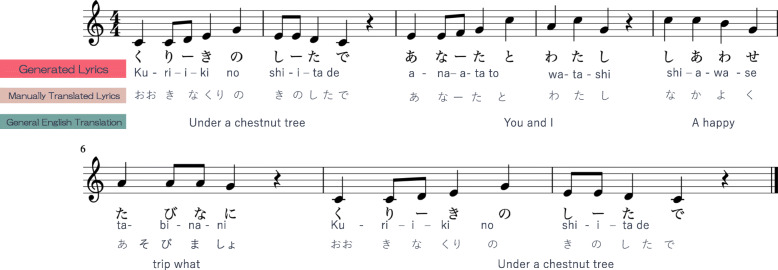
“Under the Spreading Chestnut Tree”.

Figures 6 and 7 present the results of lyrics translation. Note that the label “Generated Lyrics” denotes the lyrics generated by our algorithm, while “Manually Translated Lyrics” denotes popular lyrics translated in Japan. “General English Translation” does not represent the lyrics, but the translation of the “Generated Lyrics”.

Both results had the same lyrics as the manual Japanese translation. However, there were phrases whose meaning was difficult to understand in the translated lyrics of “Under the Spreading Chestnut Tree”. From a musical perspective, the lyrics “my fair lady”, which is the last phrase of “London Bridge”, is difficult to sing in Japanese because “my fair” is two syllables in English but four morae in Japanese (i.e., “ma i fe a”).

These results indicate that this algorithm can be used to perform lyrics translation of simple songs if users modify the lyrics in the system. In addition, the algorithm can solve Problem 1, as it can reduce the number of lyric morae by deleting words such as honorific and assertive expressions and abbreviating Japanese particles.

### Preliminary experiment

We conducted a preliminary experiment to determine the optimal combination of user interface components. This experiment was conducted to quantitatively evaluate the components of the user interface. In addition, we focused on the click count and time to improve the efficiency of lyrics transcreation. Participants were instructed to work on lyrics transcreation using the presented system. Four phrases from the song “Scarborough Fair” were selected for transcreation in this experiment.

We installed this system on a web server and prepared a private experimental environment. When a participant accessed the system, the system displayed an introductory description and then began the lyric transcreation routines. When the transcreation task was complete, the system displayed a questionnaire form and stored the user’s operation history. The operation history data recorded during the task included the position and frequency of mouse clicks, the total time spent on the task, and the combination of displayed user interfaces. We calculated the average value and standard error of the recorded values for each combination of user interfaces. In addition, we conducted t-test for all items.

We recruited 19 participants (nine males and ten females, 23 to 37 years of age; average age: 27 years) for this experiment. All participants were web engineers or designers, as we believed that participants who were familiar with web applications were appropriate for selecting the best user interface. Ten of the participants could also read scores.

Table 2 displays the classification of participants. We divided the participants according to a combination of modules for aggregation and comparison of the results.

**Table 2:** Participant classification

Participant classification	Combination	Number of participants
A0 group	A0 + B1 + C0 A0 + B1 + C1	6
A1 group	A1 + B1 + C0 A1 + B1 + C1	6
B0 group	A0 + B0 + C0 A0 + B0 + C1	7
B1 group	A0 + B1 + C0 A0 + B1 + C1	6
C0 group	A0 + B0 + C0 A0 + B1 + C0 A1 + B1 + C0	9
C1 group	A0 + B0 + C1 A0 + B1 + C1 A1 + B1 + C1	10

#### Results of lyrics translation candidates

**Fig. 8:**
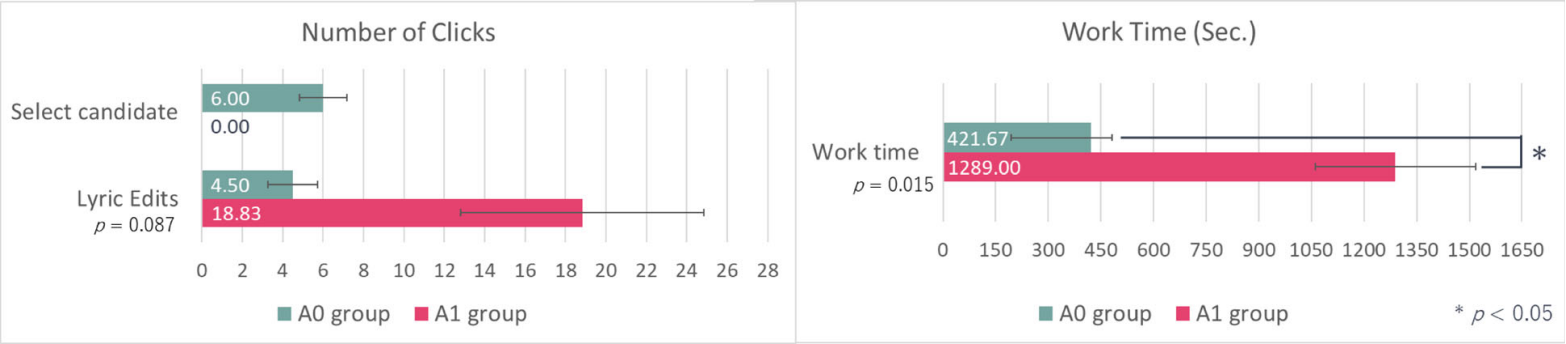
Comparison of number of clicks and work time between A0 and A1 groups

Figure 8 compares the average number of clicks, average time, and standard error of the A0 group and A1 group. As can be seen, “Select candidate” of the A1 group was 0.00, which signifies that the selection widget was not displayed in the user interface. The average number of clicks in the selection widget was 6.00 for the A0 group.

The average number of lyric edits in the text field was 4.50 for A0 group and 18.83 for the A1 group. Although no significant difference appeared for this item, it had a significant trend because the *p*-value was 0.087.

In addition, the work time for the A0 group was 421.67 s, while that for the A1 group was 1289.00 s. There was a significant difference for this item.

These results suggest that users had shorter edit and work times when using A0. Therefore, we concluded that lyrics translation candidates should be displayed in the selection widget.

#### Results of lyrics editor

**Fig. 9:**
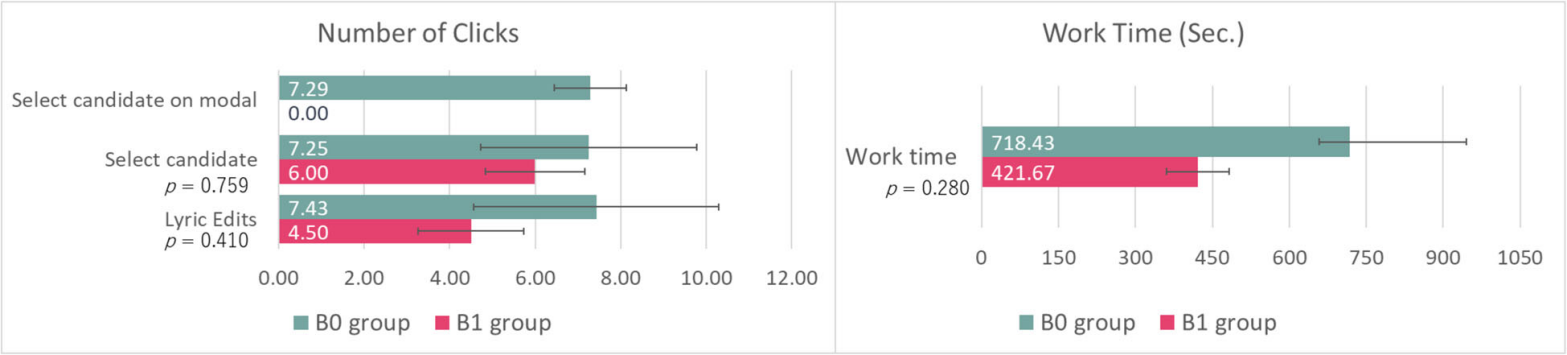
Comparison of number of clicks and work time between B0 and B1 groups

Figure 9 compares the results of the average number of clicks, average time and standard error between the B0 group and B1 group.

Here, the “Select candidate on modal interface” of the B1 group was 0.00, which signifies that the modal user interface was not displayed. The average number of clicks in the selection widget was 7.29 when displaying the modal user interface for the B0 group.

Although the participants first selected lyrics candidates from the selection widget on the modal user interface when using B0, the average number of clicks on the selection widget of the subsequent editing screen was 7.25, 0 while that of the B1 group was 6.00. The number of lyric edits in the text field was 7.43 and 4.50 for the B0 group and B1 group, respectively, while the work time was 718.43 s for the B0 group and 421.67 s for the B1 group.

There were no significant differences for any items. The B1 group appeared to be better than the B0 group in reducing the number of screen transitions because no significant differences were observed in “Select candidate” even when selecting a lyrics translation candidate on the modal user interface. Therefore, we selected B1, which provides the option to edit from the score with the translated lyrics.

#### Results of note editor

**Fig. 10:**
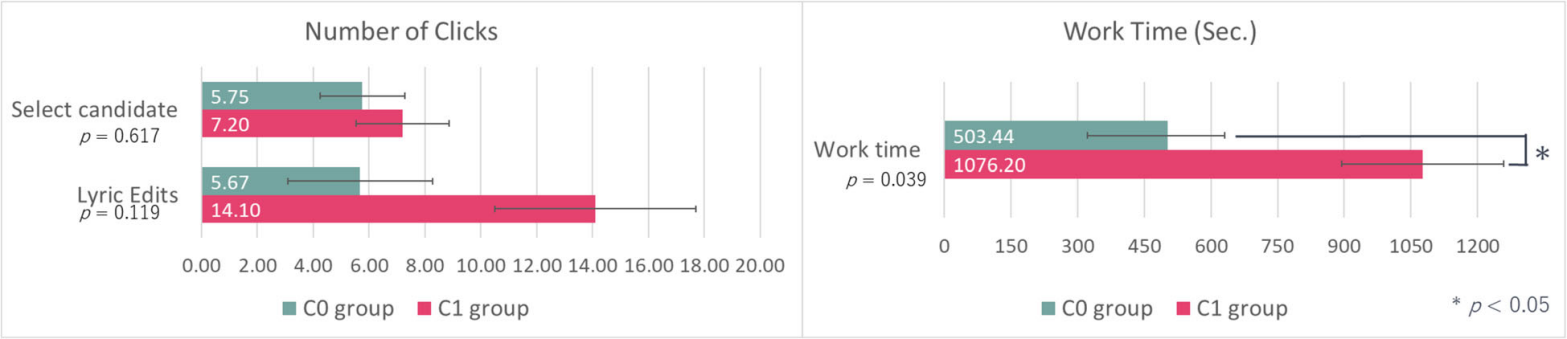
Comparison of number of clicks and work time between C0 and C1 groups

Figure 10 compares the results of the average number of clicks, average time and the standard error between the C0 group and C1 group.

The results reveal that the number of operations was smaller when using C0, and the work time for the C0 group was smaller than that of the C1 group.

There was also a significant difference for one of three items. From these results, we concluded that notes should be modified automatically.

### Discussion of preliminary experiment

We identified three directions for the development of interactive lyrics transcreation systems from the results of the preliminary experiments as follows: (1) The system suggests multiple lyrics translation candidates using the selection widget; (2) Users edit the lyrics in real time on the musical score where the temporary lyrics translation is displayed; (3) The system automatically modifies notes during lyrics transcreation.

In addition, we received the following comments about the user interface from participants:

- I want to move between phrases by clicking the bar showing the phrase at the top of the score (three participants).
- A trial listening function should be provided not only for the entire song but also for each phrase (three participants).
- I do not know the typical methodologies of lyrics transcreation, so it is preferable if this system shows a task list.
- It is helpful to display English lyrics while editing Japanese lyrics, so the Compare tab should be merged with the Edit tab.

In implementation extended after preliminary experiment section, we described our improvements of the user interface based on these comments.

### Comparative experiments

This section introduces our experiments that compare lyrics transcreation using the presented system and manual lyrics transcreation to determine which method was superior. In each case, participants were allowed to use a web search engine while transcreating lyrics, as it is common for individuals to use web search engines in everyday life. In addition, the participants were allowed to continue lyrics transcreation tasks until they were convinced with the transcreated lyrics. The section describes the procedures of the experiments for both the presented system and manual transcreation.

The procedure for lyrics transcreation using the presented system is as follows: (1) Listen to the original song and view the original lyrics; (2) Practice the manual lyric transcreation for five minutes; (3) Learn the functionality of the presented system; (4) Practice the presented system for five minutes; (5) Transcreate lyrics using the presented system; (6) Answer the questionnaire.

The following is the procedure for manual lyrics transcreation: (1) Listen to the original song and view the original lyrics; (2) Learn the functionality of the presented system; (3) Practice the presented system for five minutes; (4) Perform manual lyrics transcreation; (5) Answer the questionnaire.

We distributed two sheets to the manual transcreation participants: one sheet with the musical score and lyrics in English and a second sheet with only the musical score.

The score of “Scarborough Fair” is applied in this experiment. Also, the score of “Lavender’s Blue” is applied while demonstrating manual lyrics transcreation and explaining the presented system.

The presented system recorded the number and target of the participants’ mouse clicks. We also recorded the working time for both the presented system and manual transcreation.

A questionnaire was administered for qualitative evaluation. The participants answered relative evaluations of lyrics transcreation using the presented system and manual transcreation.

### Results

We only recruited expert participants who could read musical scores for the experiments. We instructed six participants to transcreate lyrics using the presented system. Also, we conducted the other six participants to perform manual lyrics transcreation. Nine participants were female, while three participants were male (21 to 54 years of age; average age: 29 years). We also asked them to self-evaluate their English abilities on a five-point Likert scale in the questionnaire. The average score of the participants using the presented system was 3.5, while the average score of the participants performing manual transcreation was 3.0.

It should be noted that the working time was not meaningful in this experiment because several participants gave up the manual lyric transcreation task. Therefore, the working time was not considered in this evaluation.

#### Result of system log

**Fig. 11:**
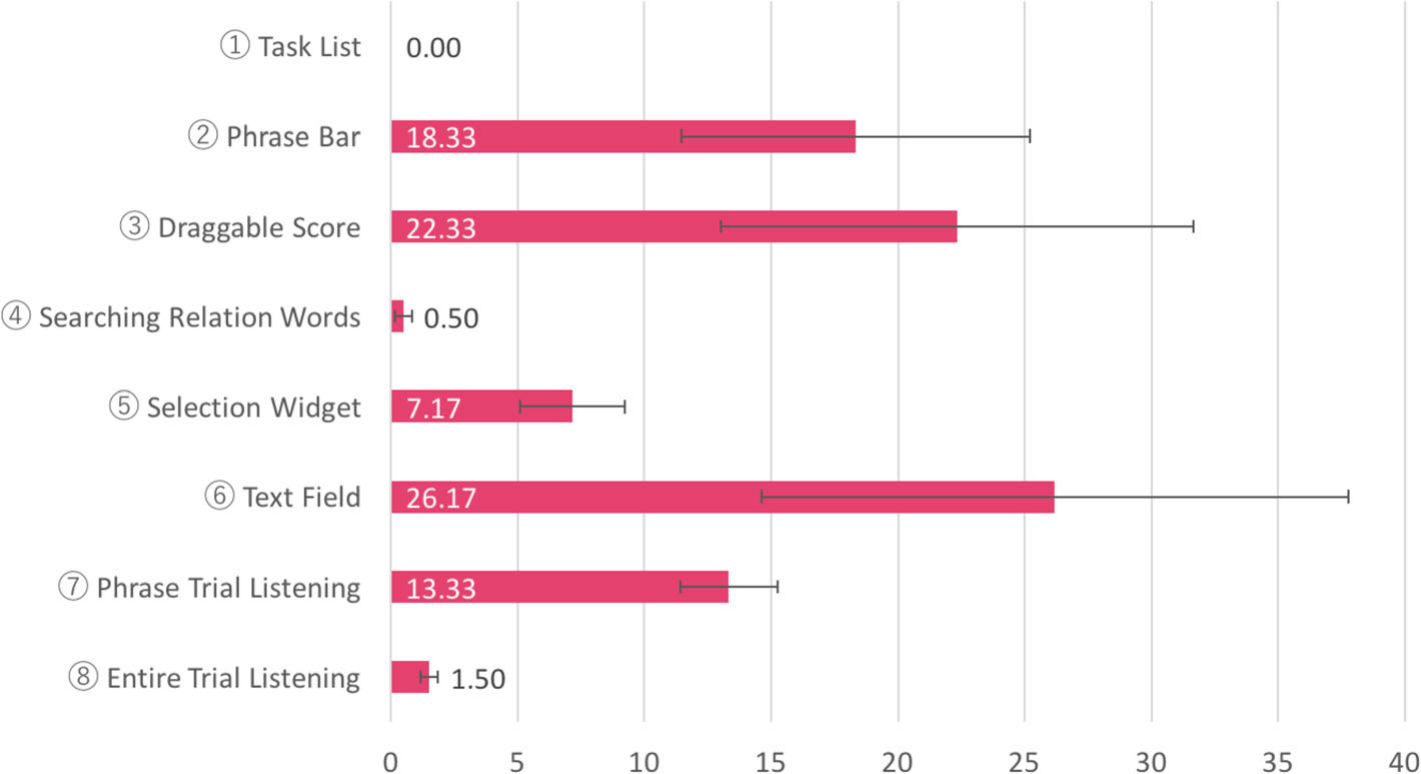
Result of the average numbers of operations and the standard error

**Fig. 12:**
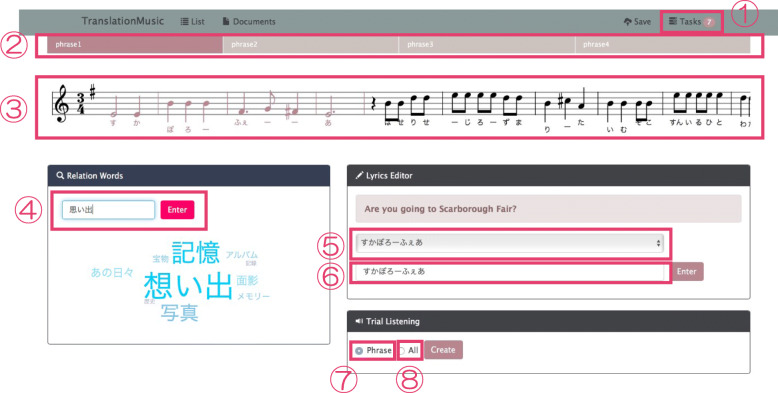
Layout of operable parts

Figure 11 displays the average number of operations performed using the presented system and the standard error. Each number on the axis corresponds to the layout of the operable parts, as illustrated in Fig. 12.

The majority of the participants did not use the task list or search for related words. We explained how to use the system before starting the experiment, and participants asked us questions while practice using the presented system. Thus, the participants understood what they were required to do and did not use the task list. In addition, we suppose that the related word search function was not used because this experiment allowed the participants to use a search engine on the web. The average number of queries to the search engine on the web of the participants using the presented system was 2.6, while that of the participants of manual transcreation was 3.0.

The average number of times the participants switched phrases by a click operation was 18.33, while the number of times the score was dragged was 22.33. These results suggest that the participants clicked the phrase bar nearly as much as the score was dragged; therefore, we concluded that the clickable phrase bar was useful.

The partial phrase listening function was clicked 12 times on average, which was more frequent than the entire trial listening function. Many participants only used the entire listening function for their final verification. This suggests that trial listening per phrase is essential for interactive modification of transcreated lyrics.

#### Comparison

**Fig. 13:**
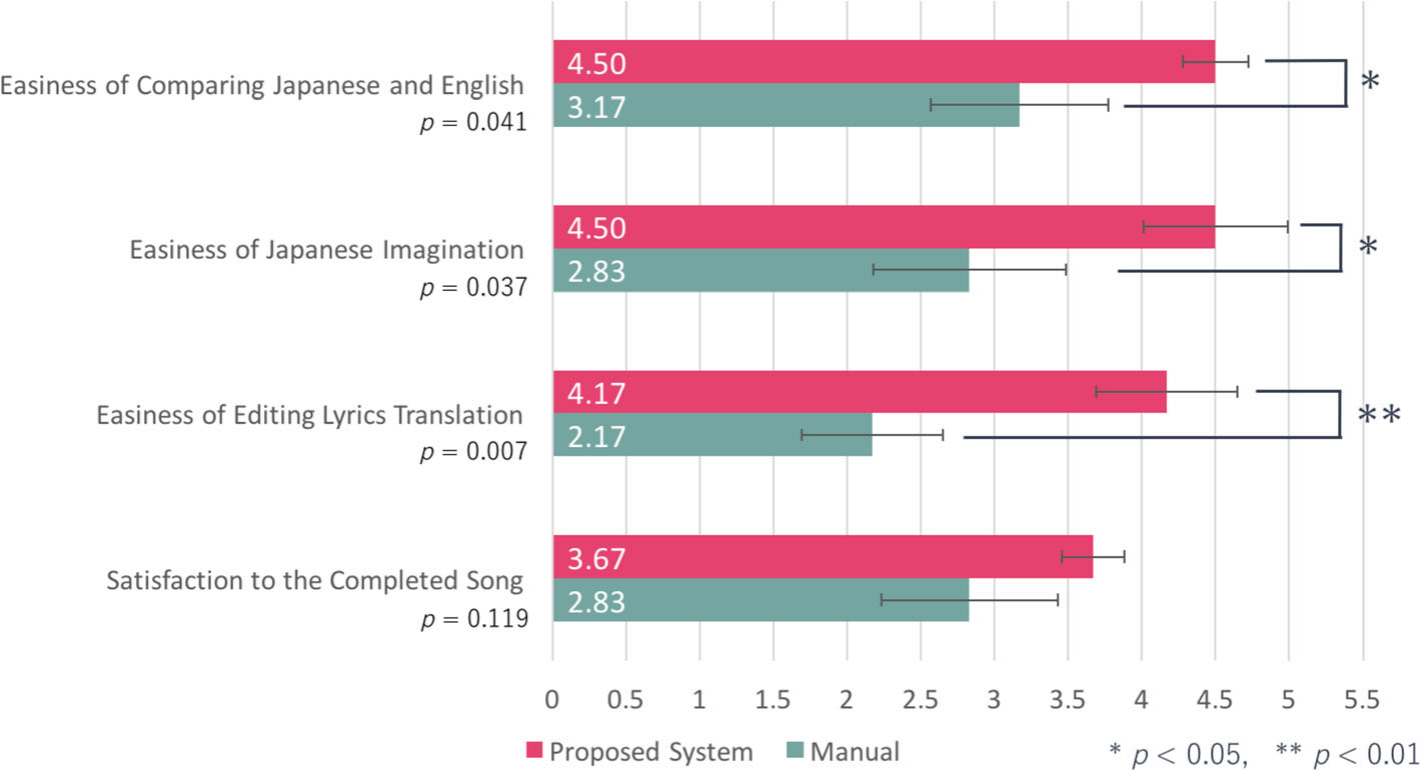
Results of comparison of the questionnaire contents [3]

This section discusses the results of the questionnaire. Here, we asked the same questions for both the presented system and the manual transcreation. Figure 13 presents the t-test, average, and standard error calculated from the answers in five-point Likert scale. The presented system archived higher scores for all questions. Also, we could observe significant differences from three of the four questions.

In terms of the ease of comparing Japanese and English lyrics, the standard error for the presented system was less than that of the manual transcreation task. We believe that lyrics can be compared more effectively using the presented system because the user interface provides a fixed presentation style. In contrast, participants in the manual task demonstrated various styles, such as writing lyrics directly on blank paper or creating lyrics by writing their transcreations underneath the English lyrics.

In terms of the ease of imagining lyrics in Japanese, several participants mentioned that it was easy to think in Japanese while using the system because the system provided lyrics candidates.

The *p*-value of the ease of editing a lyrics transcreation was 0.007, which was less than for other items. As a result, we found that it was easier to use the presented system than transcreating lyrics manually even for expert participants who could read musical scores.

The satisfaction with the completed lyrics revealed a drastic difference rather than the standard error. The participants could easily modify and were convinced by the lyrics generated using the presented system. In contrast, several participants gave up transcreating lyrics manually. As a result, these participants had lower scores for manual transcreation. The evaluation of the presented system was slightly lower than that of other questions; thus, improving satisfaction will be the focus of future work.

The *P*_*avg*_ and *P*_*med*_ values for transcreation using the presented system and manual transcreation are as follows. System: *P*_*avg*_ = 3.79 and *P*_*med*_ = 3.5; manual: *P*_*avg*_ = 3.67, *P*_*med*_ = 3.0. There was no particularly significant difference between the system and manual transcreation. Although the system provided temporary translations of *P*_*avg*_ = 4.75 and *P*_*med*_ = 5.0, we found that user edits can reduce the difference between the original melody and translated lyrics’ morae.

#### Comments of participants

The participants provided the following comments regarding the presented system and the manual transcreation.

Positive comments about the presented system are as follows:

- It was helpful to listen to the edited song in real time (two participants).
- Lyrics editing using translation candidates is useful (four participants).
- Automatic change of the note division is good.

Negative comments about the presented system are as follows:

- I could not change note division by myself (three participants).
- It seems there is no rule for musical note division because one of the algorithms is randomly applied.
- I wanted a function to listen to the original and edited songs for comparison because I had to listen to the original song on YouTube while working.

Four participants indicated that the lyrics candidate function was useful. Comments regarding the musical note editing method were divided: several participants commented that automatic edits were useful, while several other participants indicated that they wanted to edit the notes manually. Automatic musical note editing was stressful for several participants because the system applied either of the two algorithms randomly.

Positive comments regarding manual transcreation are as follows:

- It was easy to rewrite lyrics.
- Lyrics transcreations could be edited based on the English lyrics.
- It was easy to understand where the lyrics were edited.
- I could freely modify note division.
- It was good that Chinese characters could be used.

Negative comments about manual transcreation are as follows:

- It was difficult to rewrite lyrics.
- It was difficult to imagine the sound of the edited song (two participants).
- I could not match the notes to the lyrics.

These comments reveal contrasting opinions: one participant noted the ease of rewriting lyrics, while another mentioned that the task was difficult. Negative comments regarding manual transcreation included difficulty imagining the sounds, while the presented system featured the listening function. In addition, it was difficult for participants to match the musical notes to the lyrics in the manual task. However, the presented system features an automatic musical note editing function. Several participants gave up transcreating lyrics manually, whereas all participants completed transcreation using the presented system.

Participants’ comments regarding the completed songs are as follows.

Songs completed using the presented system:

- I was able to transcreate better, but I desired a greater ability to optimize the correspondence between notes and lyrics (two participants).
- The lyrics were too dense.
- The edited lyrics depended on the lyrics candidates presented by the system.
- It was easy to create good lyrics in a short time (two participants).
- I do not understand English, but it would be fun to transcreate lyrics using the system.

Songs completed by manual transcreation:

- It became a disappointing song.
- It was a literal translation, not a lyrics transcreation (three participants).
- I felt a lack of talent relative to lyrics transcreation.

One participant suggested that the lyrics transcreations in the completed song were dependent on the lyrics candidates in the presented system and lacked originality.

Several participants mentioned that the manual lyrics transcreations were similar to literal translations. In contrast, participants who used the presented system indicated that they wished that their lyrics better fit the notes. While participants of manual lyrics transcreation were struggling with literal translation, the participants using the presented system were thinking about how to make the transcreation suitable for the melody. This suggests that the presented system can solve the literal translation problem.

#### Expert review

We also interviewed two experts about our system, whose comments are as follows:

- I think this system would be very useful for translation work.
- The synthesized song can be verified if we can listen to lyrics properly in Japanese.
- The related word search function helps me, because I often wrestle with the dictionary looking for synonyms to fit the melody.
- I would like to save the lyrics as data to compare multiple transcreated lyrics candidates.
- The related word search would function better if we could look up not only related words but also synonyms.
- It would be better to be able to manually input the notes because the rhythm changes according to the transcreation.
- I would like to put multiple hiragana, such as double vowels and voiceless sounds, in a single note.

The experts provided a generally positive opinion of our system. However, the input of musical notes and the allocation of hiragana were controversial.

## Discussion

The following conclusions were drawn from the results of the comparative experiment: (1) Users felt easier to use the presented system than manual lyrics transcreation even if they could read musical scores; (2) The related word search and task list functions were not used; (3) The completed song was dependent on the lyrics candidates provided by the presented system; (4) It was difficult to optimize lyrics and musical notes even in the presented system; (5) Even if temporary translations with a large number of morae were presented by our system, the number of morae could be decreased through user edits.

The presented system can solve several problems with manual transcreation. We found that many users felt helpful with the selection of lyrics candidates, trial listening, and automatic note division.

On the other hand, we observed several problems. From the results of the number of clicks, we found that the related word search and task list functions were unnecessary. The participants used web search engines instead of the functions of the presented system when they wished to search for related words. However, the experts stated that the related word search was useful. We must thus evaluate whether the use of related words will change after we modify the representation of related words and improve the accuracy of the search.

Another problem was that the satisfaction of lyrics depends on the generation of lyrics candidates. We expected users to reflect their intent and cultural contexts in transcreation by reading a temporary translation and searching for related words. However, this is difficult if the system cannot interpret the cultural context and have it reflected in the interactive transcreation by the users. For example, four herb names appear in the lyrics of Scarborough Fair used in this experiment: “parsley, sage, rosemary, and thyme”. Although in the Western cultural context, these herbs were considered a talisman in the middle ages, none of the participants reflected this cultural context during the transcreation into Japanese lyrics. This occurred because the users could not infer the cultural context from the temporary translations and believed that the herbs only signified plants. The problem of machine translation ignoring cultural context in original languages has been previously discussed [27–29]. In the future, we would like to design functions that stimulate the creativity of users in addition to the implemented functions, such as the related word search function. It is also necessary to consider methods for incorporating the cultural context of the original lyrics into the temporary translations.

The participants wished that the presented system optimizes the correspondence between the notes and lyrics. The current implementation applies either of the two algorithms for automatic note editing. We believe that this may create a stressful situation for users because one of the algorithms is irregularly applied. Thus, we would like to archive fixed rules for the musical note editing function. In addition, further investigation is required to determine whether users edit notes freely or apply automatic note editing.

By allowing users to edit machine translations through the presented system, the number of morae can be further decreased. This suggests that the system and user collaboration can improve lyrics transcreation compared to only applying machine translation to solve Problem 1. However, the quality of the lyrics transcreated by the users should be evaluated. Watanabe et al. [30] performed a qualitative evaluation that used a crowdsourcing mechanism and quantitative evaluation involving a test set of perplexity and a line/block boundary replication task for measuring the consistency between the melody and boundaries in the generated lyrics. Dzogang et al. [31] focused on the task of detecting emotion in texts and analyzing texts as point sets in an emotional multidimensional space. We can parse lost or newly gained meaning by transcreation by analyzing emotion expressed by the lyrics and comparing the original and transcreated lyrics.

## Conclusions and future work

This paper presented the preliminary study and implementation of our visual system that supports the transcreation of lyrics written in English into Japanese. This system features the real time visualization of a user’s edits on a musical score, which helps users easily read the transcreated lyrics and melodies. We conducted a preliminary experiment with 19 participants and a comparative experiment with 12 participants. As a result, we observed that lyrics transcreation by the presented system was evaluated more positively than manual transcreation. This paper also presented an automatic lyrics transcreation algorithm. We determined that the proposed automatic lyrics translation algorithm solved Problem 1 that the number of morae typically increases when translating into Japanese for a simple song.

In future work, we plan to enhance the functionality of the presented system. Our experiment demonstrated that the related word search functions were not useful; thus, we will improve the accuracy and the interactivity of this function. In addition, we will implement automatic lyrics evaluation functions, such as presenting a lyric score points graph and advice for lyrics improvement. We expect that this new function will stimulate the creativity of users and make it easier to create transcreated lyrics without depending on lyrics candidates. Another problem is that the presented system selects either of the two algorithms randomly while matching the lyrics and melody by dividing the notes. We would like to solve the problem by adopting rules that will allow users to edit notes by simply inputting vowels or stretching bars as lyrics.

It is also an important issue to improve the algorithm of the automatic translation for candidate lyrics generation by reflecting cultural contexts. Furthermore, we plan to improve the accuracy of melody and lyric matching, such as by considering breaks in the melody and lyrics. Future experiments will also include a qualitative evaluation of completed songs.

In addition, we aim to identify solutions to Problems 2–4 in translating lyrics into Japanese.


**Additional file 1.**


## Data Availability

The dataset applied in this paper is open and it is specified in the reference.

## Abbreviations

API: Application programming interface
tf-idf: Term frequency-inverse document frequency
